# Investigation of phonon coherence and backscattering using silicon nanomeshes

**DOI:** 10.1038/ncomms14054

**Published:** 2017-01-04

**Authors:** Jaeho Lee, Woochul Lee, Geoff Wehmeyer, Scott Dhuey, Deirdre L. Olynick, Stefano Cabrini, Chris Dames, Jeffrey J. Urban, Peidong Yang

**Affiliations:** 1 Department of Chemistry, University of California, Berkeley, California 94720, USA; 2 Materials Sciences Division, Lawrence Berkeley National Laboratory, Berkeley, California 94720, USA; 3 Department of Mechanical and Aerospace Engineering, University of California, Irvine, California 92697, USA; 4 Molecular Foundry, Lawrence Berkeley National Laboratory, Berkeley, California 94720, USA; 5 Department of Mechanical Engineering, University of California, Berkeley, California 94720, USA; 6 Department of Materials Science and Engineering, University of California, Berkeley, California 94720, USA; 7 Kavli Energy NanoScience Institute, Berkeley, California 94720, USA

## Abstract

Phonons can display both wave-like and particle-like behaviour during thermal transport. While thermal transport in silicon nanomeshes has been previously interpreted by phonon wave effects due to interference with periodic structures, as well as phonon particle effects including backscattering, the dominant mechanism responsible for thermal conductivity reductions below classical predictions still remains unclear. Here we isolate the wave-related coherence effects by comparing periodic and aperiodic nanomeshes, and quantify the backscattering effect by comparing variable-pitch nanomeshes. We measure identical (within 6% uncertainty) thermal conductivities for periodic and aperiodic nanomeshes of the same average pitch, and reduced thermal conductivities for nanomeshes with smaller pitches. Ray tracing simulations support the measurement results. We conclude phonon coherence is unimportant for thermal transport in silicon nanomeshes with periodicities of 100 nm and higher and temperatures above 14 K, and phonon backscattering, as manifested in the classical size effect, is responsible for the thermal conductivity reduction.

## Abstract (web summary)

Low thermal conductivities in nanomeshes have been attributed to both wave-like and particle-like behaviour of phonons. Here, the authors use periodicity-controlled silicon nanomeshes to show that the particle backscattering effect dominates for periodicities above 100 nm and temperatures above 14 K.

Understanding phonon transport at the nanoscale is critical for a broad range of semiconductor technologies^1^ involving nanoelectronics, phase change data storage, heat-assisted magnetic recording, solid-state lighting and thermoelectric energy conversion. Past studies on the thermal conductivity (*k*) of silicon materials have substantially contributed to establishing microscopic phonon transport models^2^. Classical theories based on the Boltzmann Transport Equation (BTE) that treat phonons as incoherent particles have accurately described the size-dependent thermal conductivity in silicon thin films^3^ and high purity nanowires^4^,^5^; these *k* reductions are due to mean free path suppression from boundary scattering. However, recent *k* measurements of superlattices^6^,^7^ and of silicon nanomeshes^8^,^9^,^10^,^11^, which are thin membranes with a fabricated periodic mesh of nanoscopic holes, have called into question the validity of this particle-based BTE approach when applied to periodic nanostructures. The periodic holes in the nanomesh introduce a secondary artificial periodicity to the original lattice, potentially modifying the phonon dispersion relations and scattering from the bulk in a phononic crystal analogy to photonic crystals^12^. The resulting phononic bandgaps and reduced group velocities would lead to a lower *k* than predicted by the particle-based models. This *k* reduction is referred to as a coherence effect due to the required phase coherence of phonon waves.

While the prospect of controlling phonon waves is attractive for potential phononic systems^13^,^14^, experimental reports remain inconclusive on the relative importance of wave-based coherence effects versus particle-based boundary scattering effects in the nanomesh. Researchers have fabricated and measured *k* of silicon nanomeshes^8^,^9^,^10^,^11^,^15^,^16^,^17^,^18^ with hole periodicities ranging from 10 μm down to 34 nm. Some of these experiments reported stronger *k* reductions than that predicted by BTE theories; these results were attributed to coherence effects^8^,^9^,^16^,^19^. In contrast, computational works by Jain *et al*.^20^ and Ravichandran and Minnich^21^ concluded that some of these experimental results could indeed be explained by particle based models without considering the coherence effects.

Underlying these various interpretations are differing views of the important coherence length scales in periodic structures. When periodicities are smaller than the dominant phonon wavelengths (*λ*), which are <10 nm in silicon^22^ for temperatures *T*>10 K, the particle models can break down^23^. When length scales are larger than inelastic mean free paths (for example, due to Umklapp scattering, *Λ*_U_), coherence effects can be safely neglected. The average *Λ*_U_ in bulk silicon at room temperature is experimentally found^24^ to be ∼300 nm, and calculations^25^,^26^ suggest that phonons with *Λ*_U_ between 100 nm and 10 μm carry ∼70% of the heat at 300 K. The current question^6^,^16^,^19^,^27^ is whether phonon coherence effects are important when periodicities (here, pitch *p*) are large compared with *λ* but small compared with *Λ*_U_. For example, studies have questioned whether coherence lengths that characterize the spatial extent of the wave packet^28^,^29^ are meaningful when wave packets can undergo multiple reflections in periodic structures^30^,^31^. A common justification for applying the particle model in periodic nanostructures is that the interfacial roughness *δ* is comparable to or larger than the coherence length^31^. However, it has been recently proposed that phonon wave effects must be considered at room temperature even with disordered interfacial length scales *δ*≈1 nm in superlattices^6^ or surface roughness *δ*≈2.5 nm in silicon nanomeshes^16^.

An alternate, purely particle-based, explanation for the measured *k* reduction in the nanomesh is the phonon backscattering effect^21^,^32^. As an illustration of backscattering, first consider an array of parallel nanowires, for example, Fig. 1b (right). The backscattering concept predicts that adding lateral bridging necks linking the nanowires into a nanomesh (Fig. 1b (left)) would reduce *k* because ballistic phonons are more likely to be scattered backwards when colliding with the nanomesh necks than with the nanowire walls, providing additional resistance to heat flow. This backscattering effect has recently been used to explain why *k* of a nanomesh could be lower than *k* of an equivalent nanowire array^21^ even in the absence of coherence effects.

Although there are several mechanisms proposed to explain the *k* reduction in silicon nanomeshes, possible coherence (wave) and backscattering (particle) effects are often coupled, and previous experimental studies were unable to isolate the dominant mechanism. For example, experiments that change the hole size at fixed porosity^33^,^34^ or change the neck size at fixed hole spacing^15^,^35^ modify both the particle and wave predictions for *k* in nontrivial ways.

Here we measure *k* of silicon nanomesh structures defined by electron-beam lithography to isolate these competing mechanisms of *k* reduction. By comparing nanomeshes with periodic and aperiodic holes of the same average dimension, our experimental design decouples the coherence effect from boundary scattering effects. Similarly, by controlling the hole-to-hole pitch from 1 μm to 100 nm, our experiments evaluate the phonon backscattering effect. The thermal conductivity measurements are performed on monolithic silicon devices using an established technique first developed for nanowires^4^. The measurement temperature was controlled from 325 to 14 K; these low *T* measurements are crucial because the important *λ* and *Λ*_U_ are larger at low *T*, which facilitates the observation of potential coherence and backscattering effects. We perform ray tracing simulations to rigorously capture the boundary scattering required for particle model *k* predictions. By comparing experimental results between samples and against particle model predictions, we show that coherence effects are not necessary to describe thermal transport in the regime where the nanomesh pitch is greater than *λ* but smaller than *Λ*_U_, and that the backscattering effect leads to the *k* reductions.

## Results

### Experimental design

To study phonon transport mechanisms in an unambiguous manner, we prepared thermal conductivity measurement devices with single-crystalline silicon nanomesh structures whose relevant dimensions are controlled using high-resolution electron-beam lithography. The nanomesh dimensions were designed to accomplish two sets of comparison schemes.

**Figure 1:**
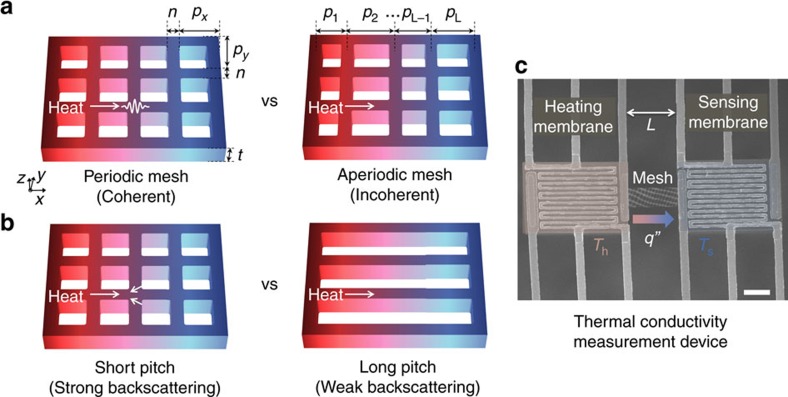
Experimental designs. Design of silicon nanomeshes to investigate phonon wave coherence and particle backscattering effects, and the experimental platform. (**a**) Coherence effects are isolated by comparing periodic and aperiodic nanomeshes with identical thickness (*t*), neck size (*n*), transverse pitch (*p*_*y*_) and average pitch along the direction of heat flow *(p*_*x*_), while varying the aperiodic individual pitches *(p*_*1*_*, p*_*2*_*,…*) by up to ±20% around the nominal *p*_*x*_ =100 nm. Coherence effects would be sensitive to the periodicity variations, while the boundary scattering is not. (**b**) The backscattering effect is quantified by comparing short and long pitch silicon nanomeshes with fixed *p*_*y,*_ *n* and *t,* while varying *p*_*x*_ between 100 nm and 1 μm for different samples. The bridging necks in the short pitch nanomeshes increase the phonon backscattering as compared with the long pitch nanomeshes. Colour gradients depicting temperature gradients are illustrative in **a**,**b**. (**c**) SEM image of microfabricated suspended heater/thermometer platforms integrated with a silicon nanomesh structure of length *L*. The thermal conductivity *k* is obtained using the temperature measurements on the heating (*T*_h_) and sensing membranes (*T*_s_) and the heat flux *q”*. Scale bar, 10 μm.

The first set of experiments tests for coherence effects by comparing nanomeshes with periodic and aperiodic holes of the same average dimension (Fig. 1a). A periodic nanomesh of 100 nm pitch *p*_*x*_ was compared with an aperiodic nanomesh of variable *p*_*x*_ ranging from 80 to 120 nm along the direction of heat flow 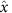. The aperiodic nanomesh was constructed to break the periodicity yet have the same porosity and average *p*_*x*_ of 100 nm. Other dimensions including the transverse pitch *p*_*y*_, neck between holes *n*, and membrane thickness *t* were kept the same between the nanomesh structures. The periodic and aperiodic nanomeshes are subject to very similar particle boundary scattering. However, coherence effects would be disrupted by the aperiodicity, as recently shown for acoustic wave propagation in the nanomesh^36^ and phonon transport in superlattices^37^. Possible effects of phonon confinement in the 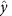 direction would also be the same between these periodic and aperiodic structures. Because the only difference between the two nanomesh structures is the periodicity in the direction of heat flow, comparing *k* provides information on the relative importance of coherence effects compared with boundary scattering effects.

The second set of experiments probes phonon backscattering by varying *p*_*x*_ from 1 μm to 100 nm (Fig. 1b), again with fixed *p*_*y*_*, n* and *t*. In the limit of large *p*_*x*_, the nanomesh resembles a nanowire array; here, *p*_*x*_ was limited to 1 μm due to mechanical instability of the released devices. Comparing *k* of samples with different *p*_*x*_ directly tests the backscattering concept by controlling the number of possible backscattering centers at the bridging necks.

Both comparison schemes require careful nanomesh fabrication. While *p*_*x*_ was varied deterministically for comparison, *n* (typically 40±5 nm s.d. between samples), *t* (typically 80±10 nm) and *p*_*y*_ (100±5 nm) were kept constant and verified throughout the fabrication processes. The thickness was chosen to maximize the chance of observing phononic crystal effects, as previous calculations^38^ showed that the membrane thickness needs to be on the order of the hole periodicity to create an optimally large forbidden band. The nanomesh patterns were transferred to monolithic silicon devices using a combination of electron-beam lithography and dry etching processes, which enabled high-resolution mesh patterns and anisotropic etching profiles with surface roughness so small as to be undetectable by scanning electron microscopy. The relevant roughness scale *δ* in the silicon nanomesh is the surface roughness, originating from the fabrication processing. The surface roughness of the nanomesh was not observable in scanning electron microscopy (SEM) images, indicating that the surface roughness *δ*<10 nm. The details of fabrication processes can be found in the Methods section.

The thermal conductivity measurement is based on a well-established thermometry technique^39^,^40^ in which microfabricated structures are suspended in vacuum to force a heat flow across a thin membrane, as shown in Fig. 1c. Two symmetric island structures are each equipped with Pt electrodes, which can act as a thermometer and a heater, and supported by slender SiN_x_ beams. Device-to-device variations are minimized by taking measurements on devices from the same chip and in close proximity. Under a high vacuum (<10^−6^ torr), one island is heated and the bridged Si nanomesh transports heat by conduction to the other island; heat losses by convection and radiation are negligible, and the monolithic silicon device minimizes thermal contact resistances^40^. Based on the measured temperature of each membrane and the amount of heat delivered to the heating island, the thermal conductance *G* of the nanomesh can be readily obtained. The measured *G* of the nanomesh is converted into the thermal conductivity *k* using the expression *GL/(wt*)=*fk*, where *L* and *w* are the length and width of the suspended silicon nanomesh and *f* is the porosity correction factor, which describes the reduction in conductance for bulk porous materials. We use finite-element method simulations with COMSOL to obtain the *f* factor for each sample, as discussed in Supplementary Note 1 and Supplementary Fig. 1. We choose to define *k* such that when all length scales are large compared with *Λ*_U_, *k* recovers the handbook value for silicon, regardless of the geometry or porosity. Using this definition, any differences in *k* for our nanomeshes are due solely to sub-continuum effects, and it is meaningful to make direct comparisons between the thermal conductivity of the nanomeshes and the thermal conductivity of fully dense nanostructures such as thin films or nanowires.

### Phonon transport modelling

The BTE particle model for the thermal conductivity under the common isotropic dispersion approximation is^41^


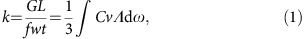


where *ω* is the phonon frequency, *C* is the volumetric modewise heat capacity, *v* is the group velocity, and *Λ* is the mean free path. We use the Born-von-Karman sine-type approximation for the bulk silicon dispersion and Matthiessen's rule: *Λ*^−1^=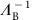
+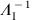
+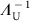 to combine boundary, impurity and Umklapp scattering, respectively (see Supplementary Note 2 and Supplementary Fig. 2 for more details regarding the BTE model). Because *n* and *t* are much smaller than the important *Λ*_U_ in silicon at our experimental temperatures^26^, the particle model predicts that boundary scattering dominates *Λ*. Analytical results for 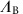 are known for simple geometries such as nanowires^42^,^43^, but *Λ*_B_ is generally unknown for complicated structures such as the nanomesh^15^,^20^. To rigorously determine *Λ*_B_ we use a ray tracing technique^44^. From the Landauer-Büttiker formalism^45^,^46^, the thermal conductance *G* is


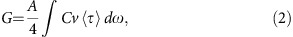


where 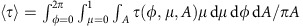 is the average transmission coefficient, *μ*=cos*θ* is the directional cosine, and *A*=*wt* is the cross-sectional area. Comparing the Landauer-Büttiker and the BTE models,


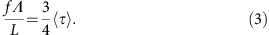


We calculate 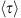 for nanomeshes with specified dimensions and surface specularities considering only boundary scattering (for details of the ray tracing implementation, see Supplementary Note 3 and Supplementary Fig. 3). To compare with experiments, the simulations use dimensions measured from SEM images, idealized as rectangular holes. We obtain the long length limit *Λ*_B_ from the slope of *L*/*Λ* against *L* , which isolates the intrinsic diffusive boundary scattering from the ballistic end effects^47^,^48^. We have validated the ray tracing code against analytical solutions for *Λ*_B_, including nanowires^44^ and cross-plane transport in diffuse superlattices^49^, as well as previously published BTE simulation results for two nanomeshes^21^ (see Supplementary Note 4 and Supplementary Fig. 4).

### Investigating coherence in periodic and aperiodic nanomeshes

**Figure 2:**
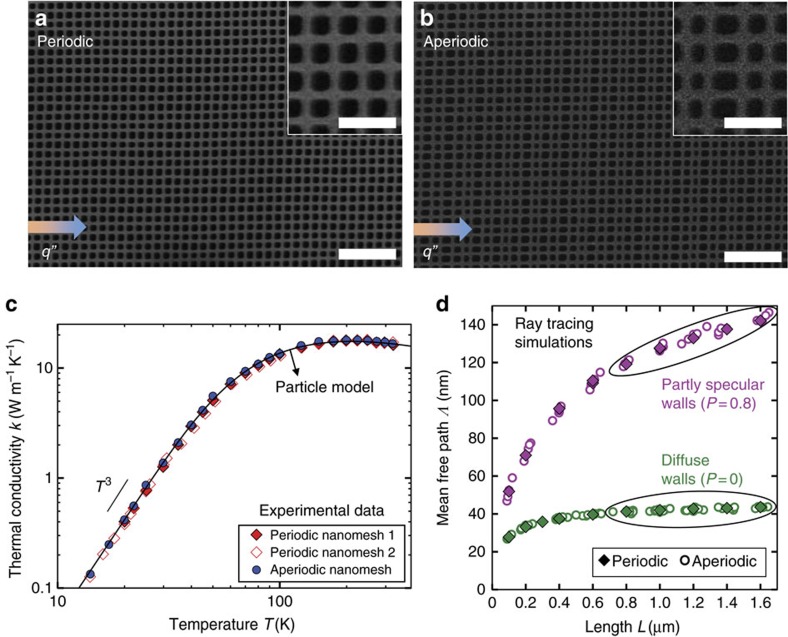
Isolating coherence effects with periodic and aperiodic nanomeshes. (**a**) SEM image of a periodic Si nanomesh with a controlled periodicity of *p*_*x*_=*p*_*y*_ =100 nm. (**b**) SEM image of an aperiodic Si nanomesh, in which the pitch in the transport direction *p*_*x*_ varies by up to ±20% (80–120 nm). The SEM images are captured after the complete device fabrication. Scale bars in **a**,**b** 200 nm (inset) and 600 nm (main). (**c**) Experimental data (points) and the BTE particle model with diffuse surfaces (line) show excellent agreement for *k*(*T*) of two periodic and one aperiodic nanomeshes. The very similar *k* between the three samples at all *T* indicate negligible coherence effects for thermal transport in silicon nanomeshes for *p*≥100 nm and *T*>14 K. (**d**) Ray tracing simulation results for the mean free path **Λ** as a function of sample length *L* considering boundary scattering with fully diffuse (*P*=0) and partly specular (*P*=0.8) surfaces show that the BTE particle model predicts equal *k* for the periodic and aperiodic meshes. The *P*=0 long-length limit, 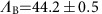 nm, was used in the particle model calculation in **c**.

Figure 2a,b show SEM images of the periodic and aperiodic nanomeshes. Image processing analysis (see Supplementary Note 5) confirmed that the periodic and aperiodic nanomeshes had equal porosities (within 1%) and average dimensions. Figure 2c shows the measured thermal conductivity as a function of temperature for three samples. The thermal conductivities of the periodic and the aperiodic samples are the same within experimental uncertainty (estimated at 6%—see Supplementary Note 6) over the entire temperature range of 14–325 K. Identical *k* from the periodic and aperiodic nanomeshes indicate that coherence effects are not important for heat transfer at these temperatures. In addition, a clear *T*^3^ trend is observed at low *T*, consistent with the classical diffuse boundary scattering theory. The measured *k* at 300 K is an order of magnitude smaller than the bulk silicon handbook value, demonstrating the importance of boundary scattering in reducing *k*. Our measured *k* is also more than a factor of two smaller than literature measurements of in-plane *k* of suspended silicon membranes of comparable thickness^2^, indicating that the mesh structuring further reduces *k* of the thin membranes.

Figure 2d shows the ray tracing simulations for the mean free path *Λ* as a function of length *L* for periodic and aperiodic nanomeshes. All surfaces were taken to be either fully diffuse (*P*=0) or partly specular (*P*=0.8). *Λ* of the periodic and the aperiodic nanomeshes are identical within the simulation uncertainty (±1%, see Supplementary Note 3) for both specularities shown here. We show that for all *P*<0.9, the periodic conductivity *k*_p_ and aperiodic conductivity *k*_ap_ remain equal within simulation error (Supplementary Note 7 and Supplementary Fig. 5). The BTE model, therefore, predicts equal conductivity for our periodic and aperiodic nanomeshes, just as observed in our experiments. In addition, the experimental results in Fig. 2c agree well with the particle model using the *P*=0 boundary scattering value. To find the impurity scattering rates required for the particle model (which have only a mild influence even at high temperature), we performed a best fit to the periodic and aperiodic experimental data (see Supplementary Note 2), which is shown in Fig. 2c as the particle model.

Because the experimental and computational results show 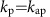, we conclude that coherence effects are not important for understanding thermal transport in silicon nanomesh structures with periodicities down to 100 nm and *T* down to 14 K. More generally, this experiment indicates that the wave nature of phonons does not need to be considered to describe transport in the regime where *λ*<<*p*<<*Λ*_U_ and *λ*∼*δ*.

### Investigating backscattering in variable-pitch nanomeshes

**Figure 3:**
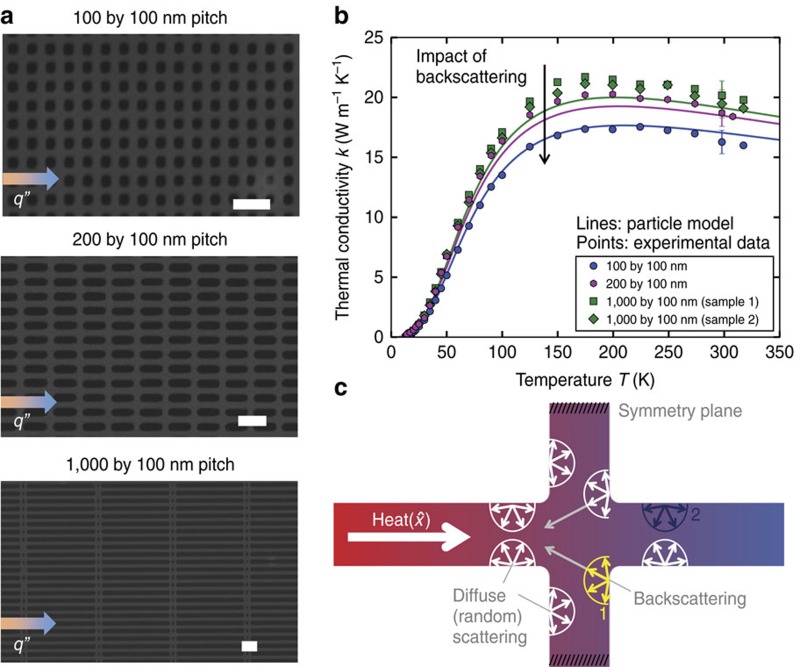
Investigating backscattering effects with variable-pitch nanomeshes. (**a**) SEM images of silicon nanomeshes with varying pitch size (100 nm–1 μm) along the direction of heat flux (*q”*). Scale bars, 200 nm. (**b**) Experimental results and particle model predictions for *k(T)* of four samples show that decreasing the pitch decreases *k*, as predicted by the backscattering effect. Error bars represent the experimental uncertainty derived using propagation of error (for details see Supplementary Note 6). The particle model contains no free parameters, and all surfaces are fully diffuse. (**c**) Illustration of backscattering for diffuse surfaces. The neck intersection backscatters a larger percentage of incident phonons that the nanowire-like boundaries parallel to the global *q”*, which leads to increased backscattering from short pitch nanomeshes. For example, 100% of the phonons are backscattered at point 1 (indicated in yellow) while the backscattering percentage is only 50% at point 2 (indicated in dark blue).

Figure 3a shows SEM images of silicon nanomeshes with pitches *p*_*x*_ by *p*_*y*_ of 100 by 100 nm, 200 by 100 nm and 1,000 by 100 nm, with similar *n* and *t* as in Fig. 2. The measured thermal conductivity *k(T)* for the corresponding nanomeshes is shown in Fig. 3b. For all *T*, *k* decreases as the aspect ratio *p*_*x*_*/p*_*y*_ decreases, as predicted by the backscattering concept. Significantly, this geometric dependence is in contrast to the bulk theory prediction of geometry-independent *k (*recall that *k* has already been corrected for bulk porosity effects using the finite-element method *f* factors). Figure 3b also shows the BTE predictions for *k*(*T*) of the nanomeshes using ray tracing results with diffuse surfaces. The particle model is in good agreement with the experimental data without using additional fitting parameters; as explained in Supplementary Note 2, the model dispersion relation inputs and Umklapp scattering rate were fit to bulk literature data^26^, the impurity scattering rate was fit to the experimental data in Fig. 2c, and the boundary scattering mean free path is calculated for each nanomesh using ray tracing.

**Figure 4:**
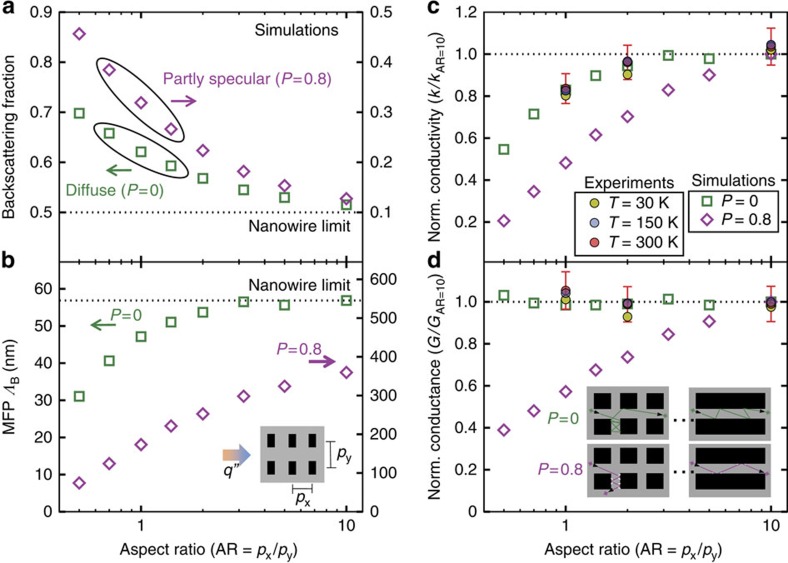
Influence of backscattering. Ray tracing simulations investigate phonon backscattering for nanomesh structures with varying pitch aspect ratios AR=*p*_*x*_/*p*_*y*_, comparing diffuse (*P*=0) and partly specular (*P*=0.8) surfaces. (**a**) Increasing the aspect ratio decreases the backscattering fraction, which quantifies the backscattering effect illustrated in Fig. 3c. For large aspect ratios, the backscattering fraction approaches the nanowire limit of (1−*P*)/2 (dashed line). (**b**) Consistent with the backscattering effect mechanism, the boundary scattering mean free path *Λ*_B_ decreases as the backscattering increases. (**c**) Normalized conductivity *k/k*_AR=10_ from ray tracing simulations (empty points) and experiments at three different temperatures (filled points, taken from Fig. 3b) show decreasing *k* with increased backscattering (that is, with smaller AR). (**d**) The normalized conductance *G/G*_AR=10_ is surprisingly independent of aspect ratio for diffuse surfaces, which we attribute to a multiple backscattering effect (inset, green). If the surfaces are partially specular, the backscattering does reduce *G*. Panels **c**,**d** show that the experimental normalized conductivity and conductance results are consistent with diffuse scattering simulations. Error bars represent the experimental uncertainty derived using propagation of errors (for details see Supplementary Note 6).

We now dive deeper into the backscattering effect illustrated in Fig. 3c with additional ray tracing simulations. We define backscattering as a boundary scattering event that changes the 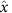 velocity component from positive to negative for a phonon originally emitted from the hot terminal, where 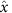 is aligned along the global temperature gradient. Backscattering reflects these phonons travelling in 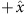 back towards the hot terminal in 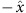 , providing resistance to heat transfer (see Supplementary Note 8 for further discussion of the backscattering definition). Figure 4a plots the fraction of boundary scattering events resulting in backscattering for phonons emitted from the hot terminal travelling in 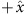 for meshes with varying *p*_*x*_, fixed *p*_*y*_=100 nm, *n*=45 nm, *t*=60 nm, and either *P*=0 (left axis) or *P*=0.8 (right axis). Decreasing the aspect ratio AR=*p*_*x*_/*p*_*y*_ increases the backscattering fraction, as expected from the argument of Fig. 3c, and for large aspect ratios the backscattering fraction approaches the nanowire limit of (1−*P*)/2. Figure 4b shows how the resulting long-length boundary scattering mean free path *Λ*_B_ decreases as the backscattered fraction increases. For diffuse scattering, AR>5 nanomeshes have very similar *Λ*_B_ to an equivalent diffuse nanowire of rectangular cross section *n* × *t* (for further comparisons between nanomeshes and nanowires, see Supplementary Notes 9 and 10, Supplementary Fig. 6, and Supplementary Tables 1–3). However, the *Λ*_B_ for partly specular nanomeshes remains below the equivalent partly specular nanowire limit, indicating that the increased backscattering in partly specular nanomeshes is still important for thermal transport even at AR=10.

We compare the ray tracing results with experiments in Fig. 4c, where the normalized conductivity *k*/*k*_AR*=10*_ from ray tracing and normalized experimental values from Fig. 3b at three temperatures show good agreement with the diffuse scattering predictions. Likewise, we plot a normalized conductance *G*/*G*_AR=10_ in Fig. 4d for both ray tracing simulation and experimental results. Surprisingly, the experiments and the ray tracing results for diffuse scattering show that *G* is essentially independent of the aspect ratio and backscattering fraction. We attribute this new observation to the effects of multiple backscattering (see Supplementary Note 11 and Supplementary Fig. 7 for additional discussion). After multiple diffuse scatterings inside the bridging necks, the phonon has no preferential direction when exiting the bridging necks, exactly as if the phonon had scattered off a physical diffuse surface of the long-pitch nanomesh (see inset of Fig. 4d). For *P*=0.8, the high-AR nanomesh has less diffuse scattering to randomize the phonon directions, and adding the bridging necks does cause a reduction in *G* as well as a reduction in *k*.

## Discussion

Previous experiments probing phonon wave effects have modified the mesh in ways that also changed the BTE particle model prediction. For example, as discussed in Supplementary Note 12 and Supplementary Fig. 8, we perform ray tracing simulations for silicon nanomeshes from a previously reported data set^16^ and find that those measurements can be satisfactorily explained by the particle BTE model without any appeal to coherence effects. For our experimental design, the particle model predicts equal conductivity for the periodic and aperiodic nanomeshes, while coherence effects would cause the conductivities to differ due to the disrupted periodicity. The carefully designed experiments, combined with supporting calculations based on the particle boundary scattering for the actual three-dimensional silicon nanomesh geometries used, allow us to conclude that the coherence effect is not important for silicon nanomeshes with periodicities of 100 nm and greater and at temperature above 14 K. We can further infer that phonon wave effects are not important for thermal transport in nanostructures when periodic length scales are large compared with the dominant phonon wavelengths.

This work and several others in the literature have compared thermal transport in nanomeshes to equivalent nanowires. Several works^10^,^21^ have concluded that the apparent nanomesh conductivity *kf* is smaller than *k*_nw_ of a nanowire with a similar rectangular cross section *n* × *t*. However, such comparisons do not account for the bulk effect of the mesh porosity factor *f*. Other measurements^8^,^35^ have indicated that even when the porosity factor is accounted for, the nanomesh conductivity *k* remains smaller than the nanowire conductivity *k*_nw_. Within the coherent phonon picture, this reduction has been attributed to a smaller group velocity arising from the modified phonon dispersion. On the other hand, within the BTE particle model, the reduction in *k* has been attributed to greater phonon backscattering off the mesh holes facing the direction of transport^21^. Our results rule out significant coherence effects, while concluding that the phonon backscattering mechanism reduces *k* (20% reduction for AR=1 versus AR=10 at 300 K).

In summary, we fabricated periodic and aperiodic silicon nanomeshes with a pitch down to 100 nm using electron-beam lithography and investigated thermal transport in the temperature range from 14 K to 325 K. Our experimental results show that periodic and aperiodic silicon nanomeshes yield identical (within ±6% experimental uncertainty) thermal conductivity, indicating the wave nature of phonons is not important in the regime where *λ*<<*p*<<*Λ*_U_ and *λ*∼*δ*. The experiments measuring *k* of nanomeshes with different pitches show that increasing the number of bridging necks decreases *k,* as predicted by the phonon backscattering effect. Simulations using a ray tracing technique provide rigorous particle model predictions for these complex nanostructures, successfully explaining all experimental results without invoking coherence effects. The insights obtained from this work will be valuable in understanding phonon transport in complicated nanostructured geometries and evaluating possibilities of future phononic applications.

## Methods

### Device fabrication

The device fabrication started with 4″ silicon-on-insulator wafers that were commercially available (vendor: SOITEC). The silicon-on-insulator wafers consisted of a device layer that was lightly doped p-type single-crystalline silicon with resistivity of 14–22 Ω-cm, carrier concentration of 10^15^ cm^−3^, (100) crystal orientation, and thickness of 340±6 nm. The buried oxide layer was 1,000±22.5 nm thick, and the handle wafer was 450±10 μm thick. First, the overall silicon membrane dimensions (width, length and thickness) were defined by photolithography and a timed deep reactive ion etching process. A 300 nm-thick low-stress SiN_x_ layer was then deposited by chemical vapor deposition and patterned by reactive ion etching as mechanical support structures. Metallic layers of 40 nm Pt and 2 nm Cr were then sputtered and patterned by reactive ion etching as electrode structures. The on-substrate devices were diced into small chips of 9.4 × 9.4 mm for further processing that includes nanomesh patterning and substrate releasing. Each chip consisted of 72 devices of varying silicon membrane width and length. The microfabrication details of monolithic silicon devices can be found in our previous publications^40^.

The silicon nanomeshes were patterned using hydrogen silsesquioxane (HSQ) as the e-beam lithography resist for its high resolution and straight line patterning capabilities^50^. An optimal development process using 1% NaOH with 4% by weight NaCl for 4 min has been used to provide high contrast. The thickness of the HSQ layers was controlled (20–50 nm) thick enough to stay uniform on the silicon membrane and thin enough to yield nanoscale patterns. Before the HSQ deposition, the chips were cleaned by sonication in acetone bath and plasma descumming processes, and Auger Electron Spectroscopy had confirmed reductions of carbon and nitrogen peaks upon the cleaning process. After the removal of surface defects, the HSQ layer was spin coated on the monolithic silicon devices on each chip and then exposed with optimal dose rates (∼17 mC cm^−2^). The HSQ patterns exposed with high dose rates have excellent etch selectivity, which enables silicon etching without involving another etch masking material such as chromium. Using a VB300 Electron Beam Lithography System, nanomesh dimensions of varying *p*_*x*_*, p*_*y*_ and *n* were obtained. The aperiodic nanomeshes were constructed by repeating a sequence of 80, 120, 90, 110, 80, 100, 120, 80, 110, 90, 120 and 100 nm. The 20% pitch variation was kept small enough to avoid fabrication variations in lithography and etching processes

The HSQ patterns were transferred to the silicon membranes by a dry etching process based on hydrogen bromide (HBr) chemistry using an Oxford PlasmaLab 150 Inductively Coupled Plasma Etcher. The HBr etching process allows the use of thin HSQ as the etch mask and provides highly anisotropic profiles. The controlled processing conditions, including 20 sccm of HBr, 20 W on the RF generator, 700 W on the Inductively Coupled Plasma generator and 20 degrees Celsius resulted in very straight sidewalls; the sidewall roughness is not observable in cross-sectional scanning electron microscopy that has a resolution of 10 nm. Large-scale roughness or scallops are not present because the HBr etching method does not involve a cyclic process. Further investigation using transmission electron microscopy may characterize smaller scale roughness (<10 nm), but would require sophisticated sample preparation and was not pursued here. After testing with various conditions, the optimal recipes of HSQ dose rate and HBr etching were successfully developed for creating 100 nm pitch nanomesh structures on a 100 nm thick silicon membrane, which can result in vertical trenches of an aspect ratio (thickness : neck) up to 5:1.

After the nanomesh pattern transfer, the silicon membranes were released by carefully removing surrounding materials that include the residual HSQ layer, the handle wafer and the buried oxide layer. The HSQ layer on top of the nanomesh structures was first removed by a plasma etching process based on 80 sccm of fluoroform (CHF_3_) and 4 sccm of argon (Ar). The backside of the chips were then processed with subsequent lithography and etching processes. After creating etching windows in the back, the handle wafer was removed by the deep reactive ion etching using alternating cycles of octafluorocyclobutane and sulfur hexafluoride. The buried oxide layer was removed by combinations of 80 sccm CHF_3_ with 4 sccm oxygen etching and the 80 sccm CHF_3_ with 4 sccm Ar etching processes. The remaining photoresists and surface defects were removed by dipping chips in PRS-3000 and water. The chips were gently dried using a critical point chamber.

### Data availability

The data that support the findings of this study are available from the corresponding authors upon request.

### Code availability

The custom MATLAB code developed for the ray tracing simulations is available from the corresponding author C.D. upon request.

## Additional information

**How to cite this article:** Lee, J. *et al*. Investigation of phonon coherence and backscattering using silicon nanomeshes. *Nat. Commun.* **8,** 14054 doi: 10.1038/ncomms14054 (2017).

**Publisher's note**: Springer Nature remains neutral with regard to jurisdictional claims in published maps and institutional affiliations.

## Supplementary Material

Supplementary Information Supplementary Figures, Supplementary Tables, Supplementary Notes and Supplementary References
