# Supplementary material for: Investigation of phonon coherence and backscattering using silicon nanomeshes
Source: Nat Commun. 2017 Jan 4;8:14054. doi: 10.1038/ncomms14054 (PMC5216120; doi:10.1038/ncomms14054)
Supplement: Supplementary Information — Supplementary Figures, Supplementary Tables, Supplementary Notes and Supplementary References [file ncomms14054-s1.pdf]

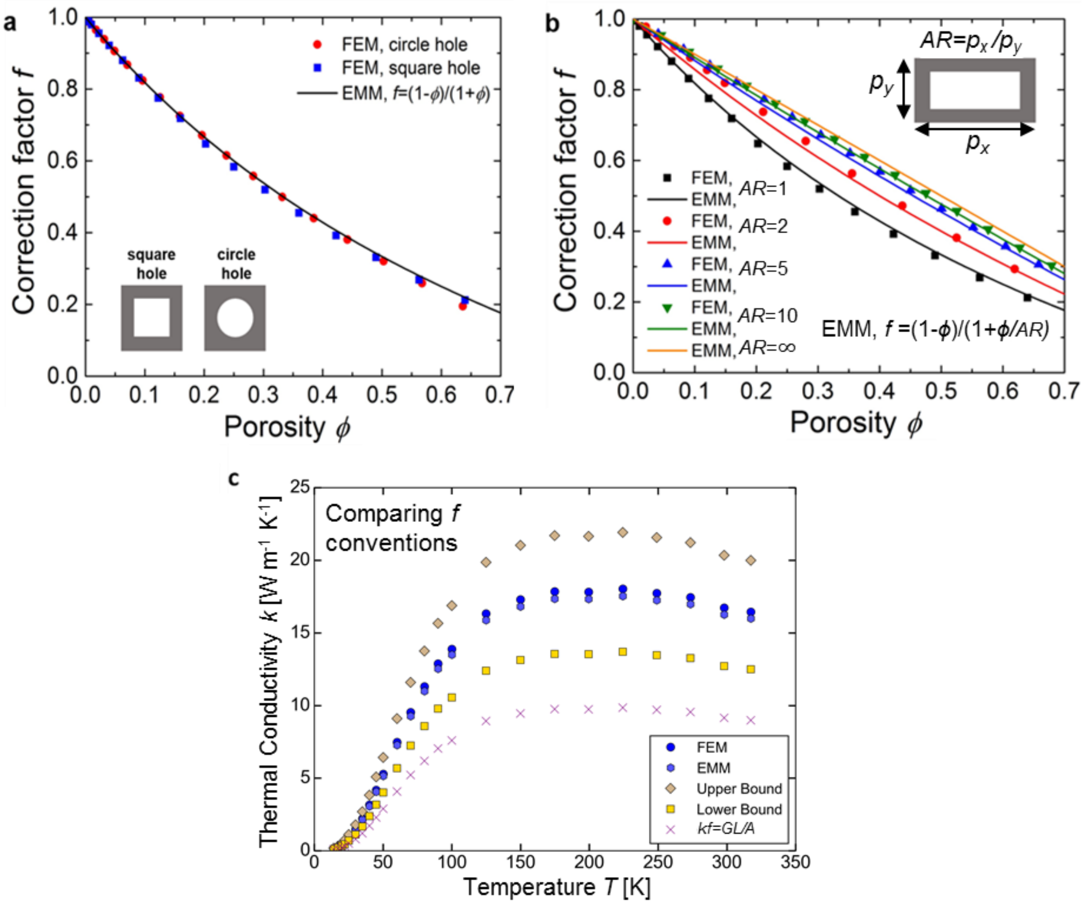

Supplementary Figure 1 | Porosity correction factor *f* and the thermal conductivity dependence on *f*. (**a**) The porosity correction factors for circular and square holes are calculated by FEM simulation using COMSOL. The correction factors for circular and square holes are almost identical to each other and in excellent agreement with the 2D Maxwell-Garnett effective medium model (EMM). (**b**) Porosity correction factors for rectangular holes of varying aspect ratios *AR* calculated by FEM simulation using COMSOL. These results are again very well explained by a 2D EMM generalized for elliptical pores. All experiments in the main text have porosity in the range $0.28 < \phi < 0.54$ and were analyzed using the FEM-derived f. (**c**) Thermal conductivity as a function of temperature using different conventions for f but the same measured G, L, and A. The use of different conventions can change the reported k values by as much as a factor of two for the periodic nanomesh (100 nm by 100 nm) investigated in this work.

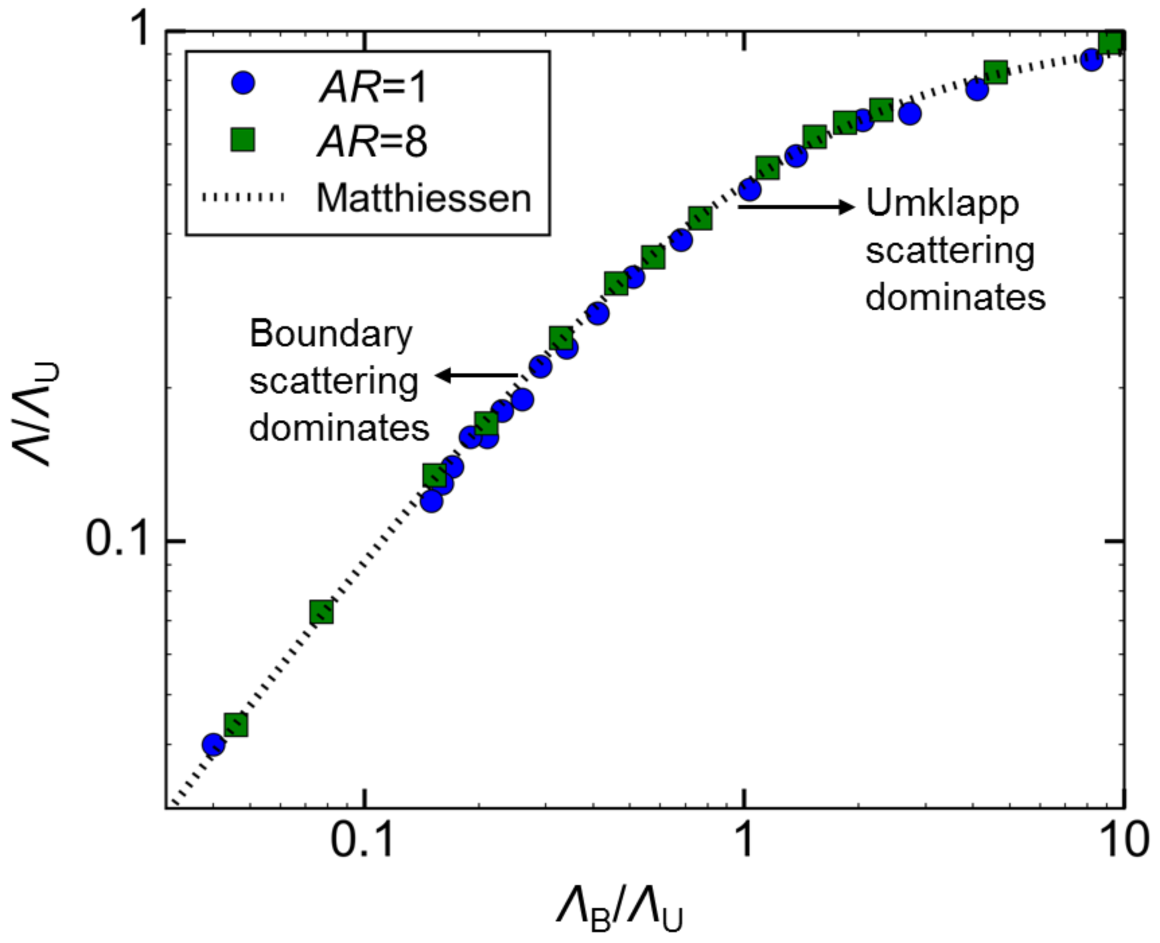

Supplementary Figure 2 | Validating Matthiessen's rule using ray tracing. Points are ray tracing simulations explicitly including both Umklapp scattering and diffuse boundary scattering, while the dashed line is the analytical Matthiessen's rule prediction for the given boundary scattering Λ_{B} and Umklapp scattering Λ_{U}. The very good agreement (7% maximum error for these dimensions of n =30 nm, t =90 nm, p =100 nm and L =0.8 μm for pitch aspect ratio *AR*=1 and *AR*=8 nanomeshes) indicates that Matthiessen's rule accurately describes the combined scattering processes for the silicon nanomeshes.

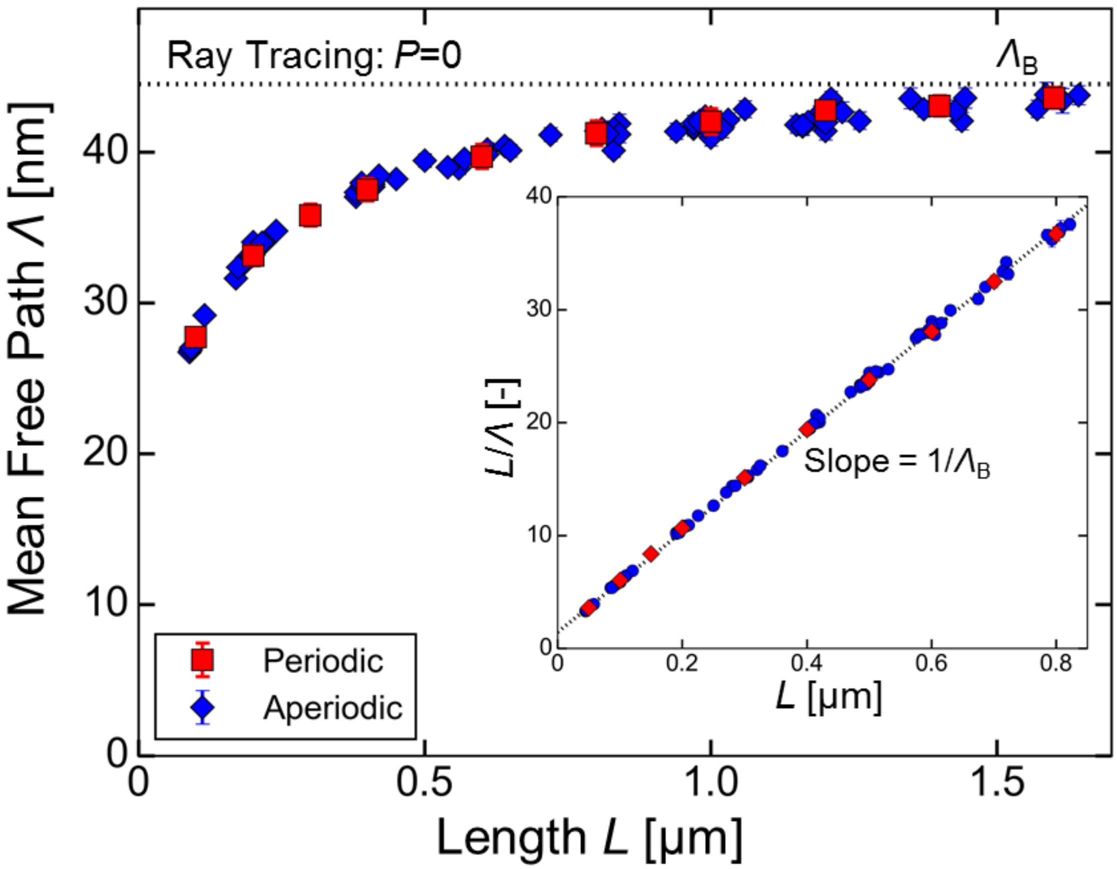

Supplementary Figure 3 | Ray tracing length convergence study. Ray tracing results reproduced from Figure 2(d) show Λ as a function of L for fully diffuse surfaces. Error bars indicate the standard error calculated as the square root of Supplementary Equation 1. The dotted line is the extrapolated long-length limit Λ_{B} , which is found from the slope of L/Λ against L (inset).

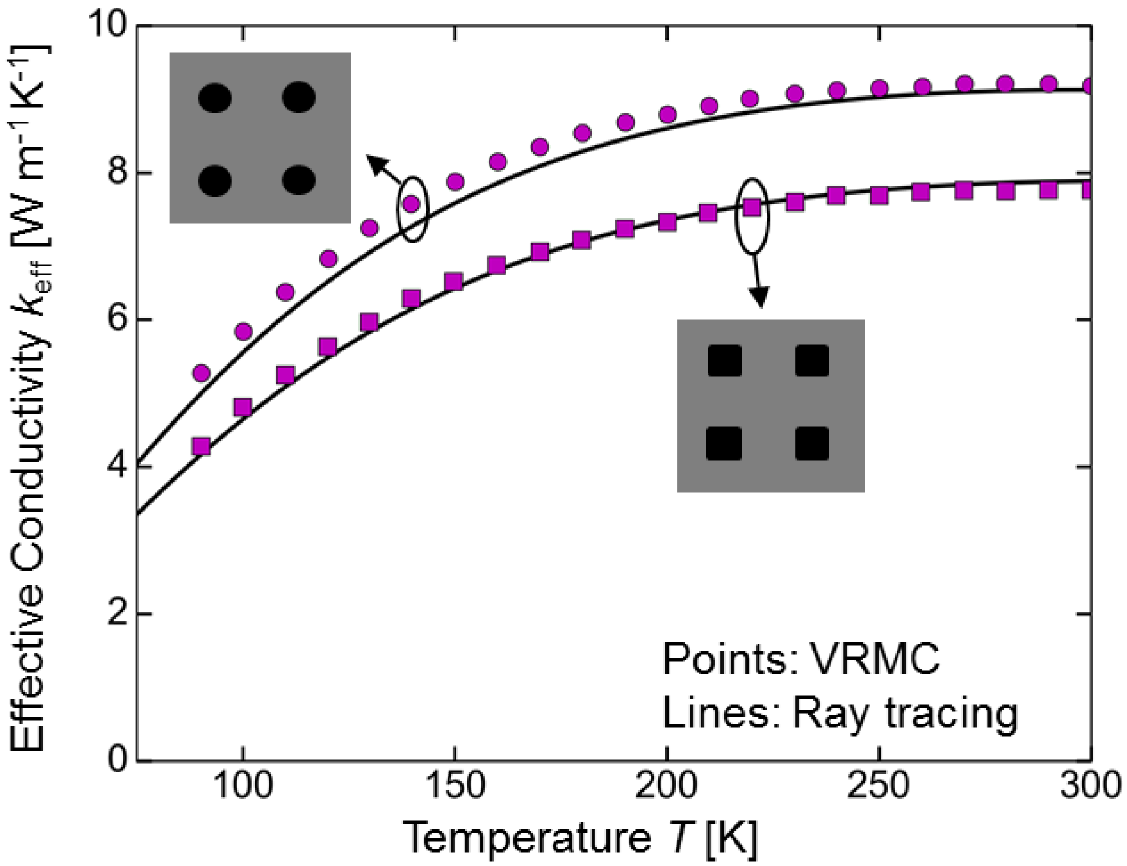

Supplementary Figure 4 | Ray tracing validation. Validating the ray tracing predictions (lines) against published variance-reduced Monte Carlo (VRMC) simulations[1] for nanomeshes with square and circular holes. The computational approaches agree within 3% for all temperatures for both the square and circular holes. For this comparison only, we use the same dispersion and bulk scattering parameters as the reference[1].

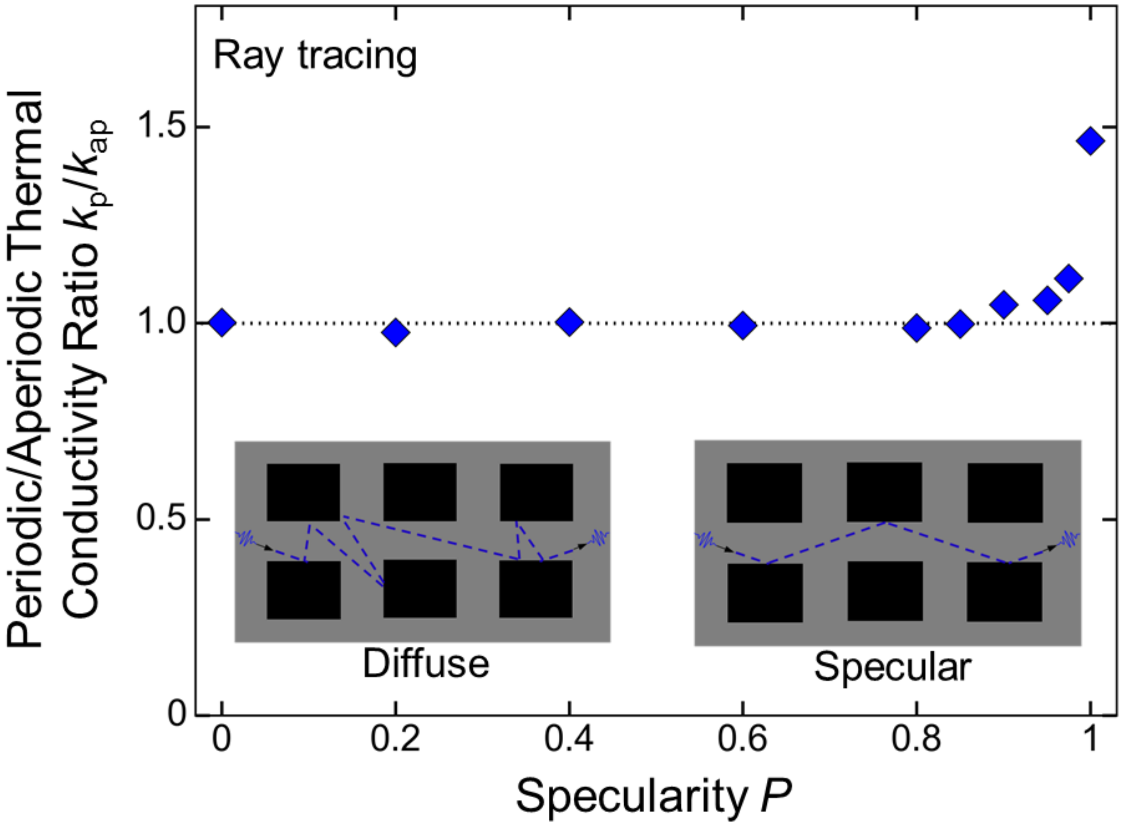

Supplementary Figure 5 | Ray tracing predictions for the periodic/aperiodic thermal conductivity ratio $k_{\mathrm{p}}/k_{\mathrm{ap}}$. Simulations were performed in the boundary scattering regime as a function of specularity *P* for nanomeshes with the same dimensions in Figure 2 of the main text and L=1.6 µm. For $P \leq 0.85$, the periodic and aperiodic nanomeshes have the same k within the error bars of the ray tracing (standard deviation ±1 %, calculated as described in Supplementary Note 3). For larger specularities, the periodic conductivity is larger than the aperiodic conductivity, which we attribute to a "critical angle" phenomena as described in Supplementary Note 7. The experiments (see main text) are modeled using the *P*=0 results.

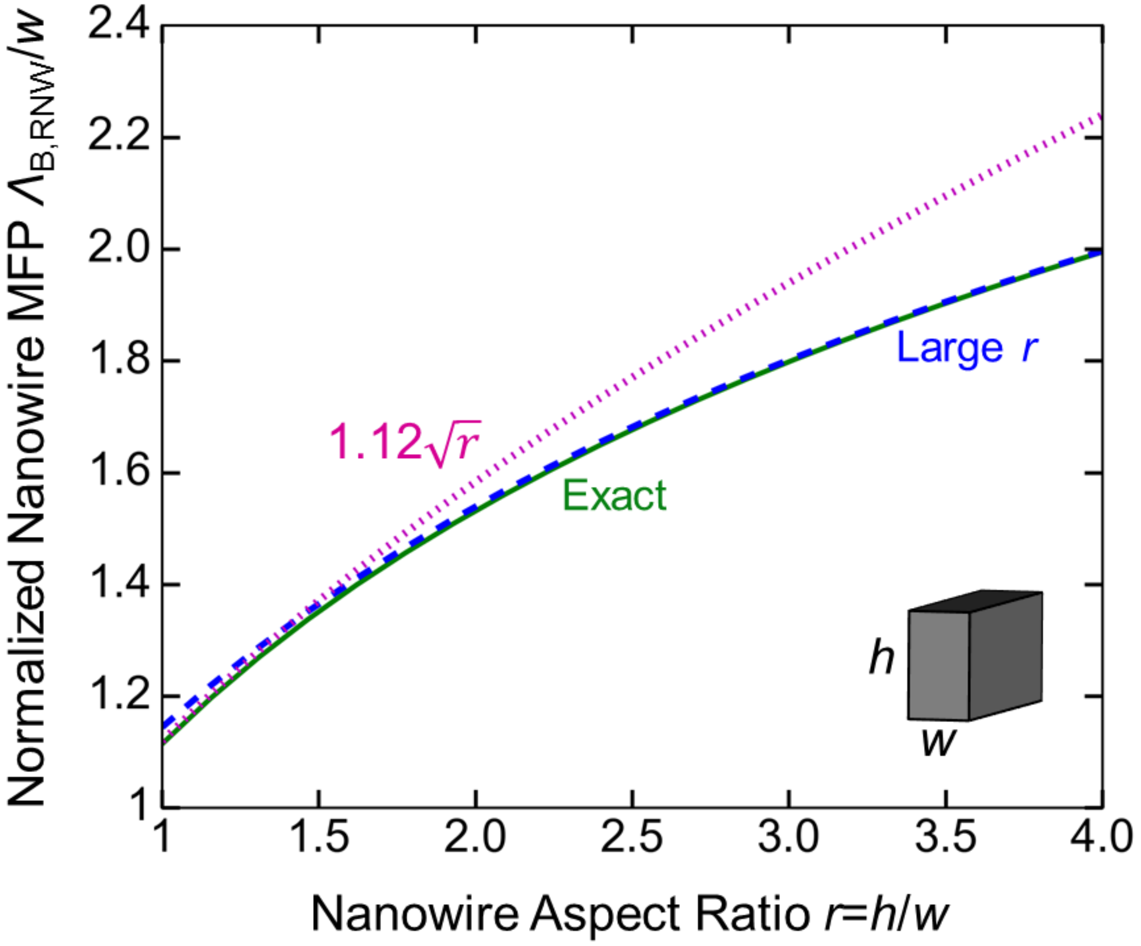

Supplementary Figure 6 | Rectangular nanowire boundary scattering. The analytical solution[2] (Supplementary Equation 5) and the limiting cases for the boundary scattering mean free path $\Lambda_{\mathrm{B,RNW}}$ of a diffuse rectangular nanowire are plotted as a function of aspect ratio $r = h/w$. The large aspect ratio limit $\Lambda_{\mathrm{B,RNW,LR}} = \frac{3w}{4}[\ln 2r + \frac{1}{3r} + \frac{1}{2}]$ (Supplementary Equation 6) is surprisingly accurate for all aspect ratios (<3% error even for 1:1 aspect ratio), while the commonly used approximation $\Lambda_{\mathrm{B,RNW}} = 1.12\sqrt{hw}$ is accurate within 5% for all $r < 2.5$.

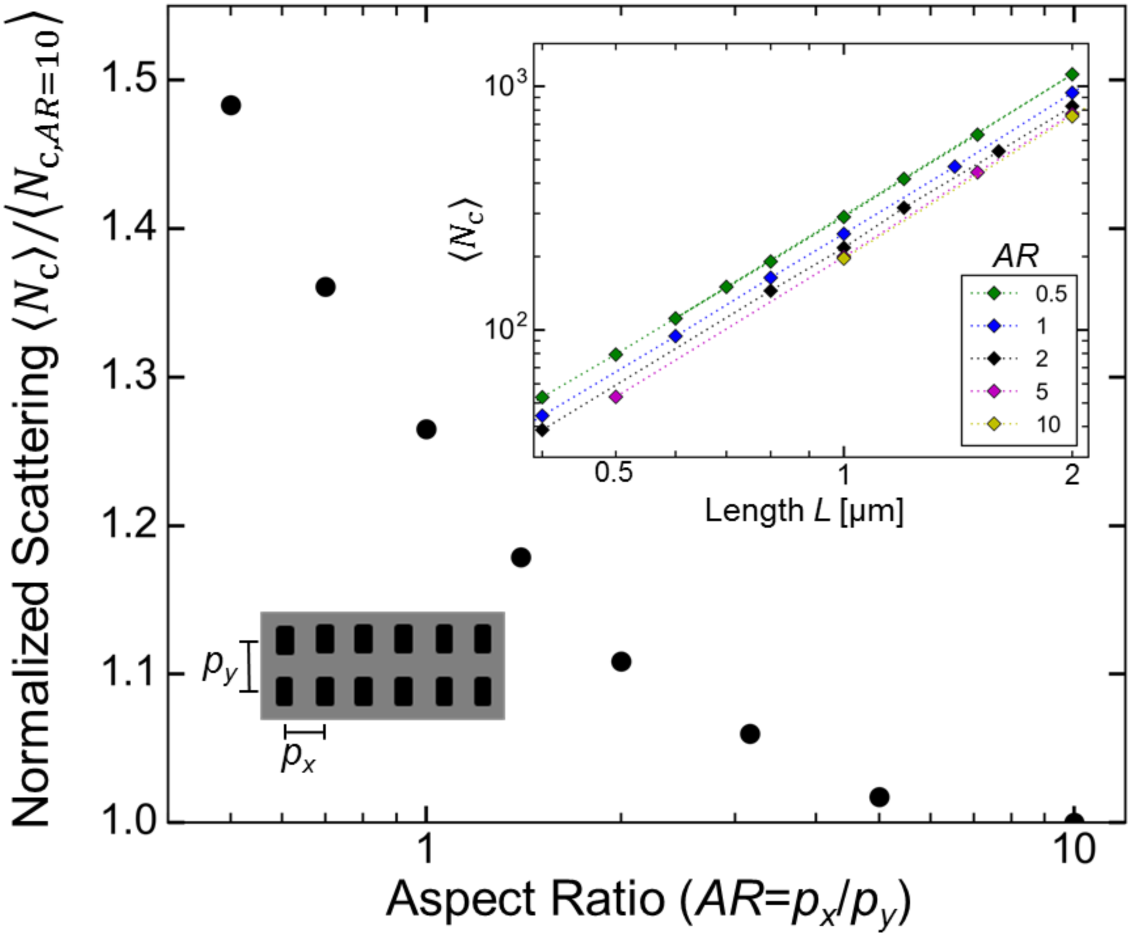

Supplementary Figure 7 | Multiple backscattering. The average number of collisions per transmitted phonon $\langle N_c \rangle$ increases with decreasing aspect ratio AR for the mesh with rectangular holes, indicating that multiple backscattering effects are important. Here n =45 nm, t = 60 nm, and p_y= 100 nm. $\langle N_c \rangle$ is normalized by the AR=10 value $\langle N_{c,AR=10} \rangle$; the inset shows that this ratio is independent of L for all aspect ratios.

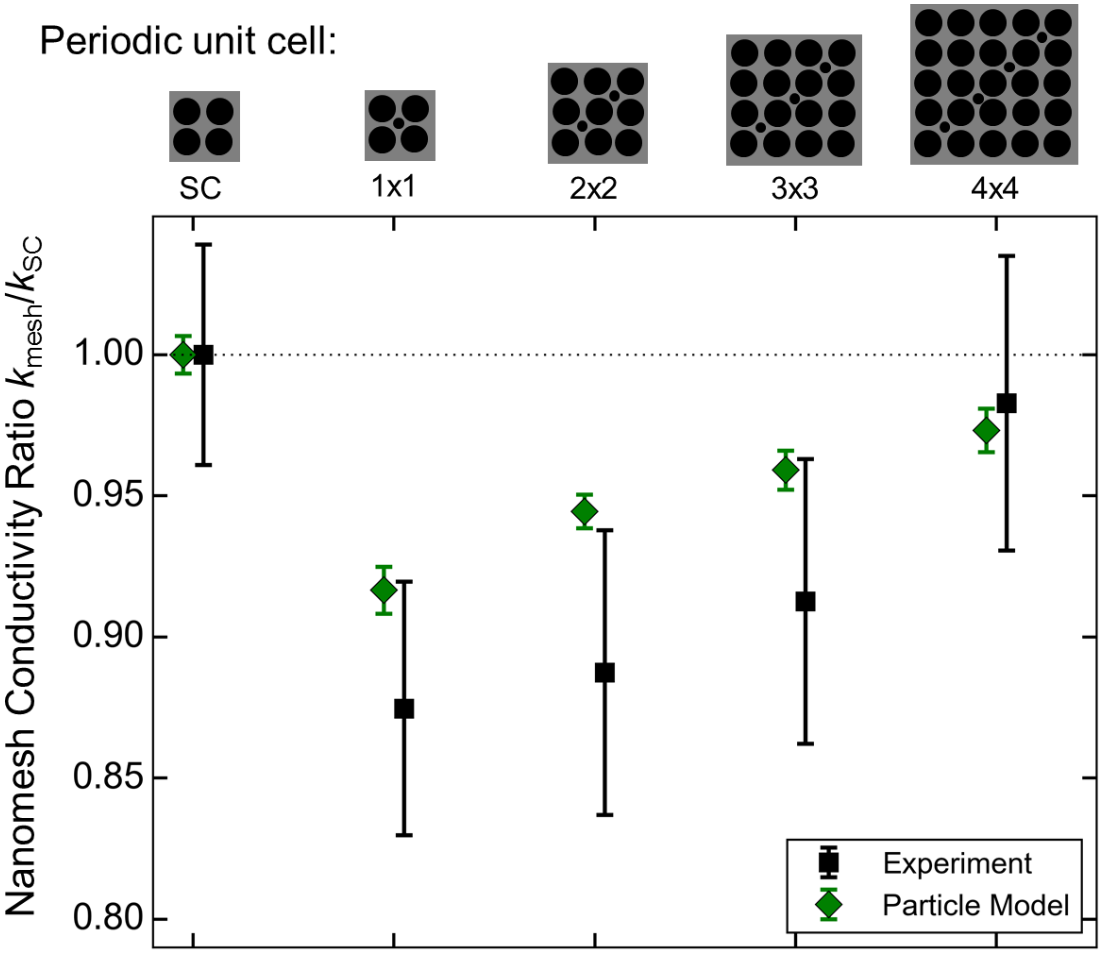

Supplementary Figure 8 | Particle model comparison with previous experiment. Particle BTE modeling results are compared to previously reported measurements[3] of thermal conductivity k_{mesh} for structures with different interpenetrating small hole arrays, normalized to the value of the original supercell k_{SC}. Ray tracing simulations predict that adding the interpenetrating small holes decreases Λ_{B} and k_{mesh}, in contrast to the previous constant-Λ_{B} assumption[3] which predicts a constant k_{mesh} (dotted line). The experimental trend is well explained by the BTE model without any modified dispersion or scattering due to coherence effects.

Supplementary Table 1 : Ray tracing results for $\Lambda_{\mathrm{B}}f$ of square hole meshes.

Neck n [nm]	Pitch p [nm]	Thickness t [nm]	$\Lambda_{\mathrm{B}}f$ [nm]	RNW error
30	100	90	15.2$\pm$0.2	6%
45	100	90	29.8$\pm$0.2	4%
45	100	60	25.3$\pm$0.3	3%
40	100	55	20.6$\pm$0.5	1%
35	100	50	16.3$\pm$0.2	1%
23	34	22	18.6$\pm$0.2	-9%
18	34	19	11.8$\pm$0.1	-8%
250	1100	366	73.2$\pm$0.3	4%

Supplementary Table 2 : Ray tracing results for $\Lambda_{\mathrm{B}}f$ of rectangular hole meshes.

Neck n [nm]	Pitch in y, p_{y} [nm]	Pitch in x, p_{x} [nm]	Thickness t [nm]	$\Lambda_{\mathrm{B}}f$ [nm]	RNW error
30	100	35	90	13.8$\pm$0.3	15%
30	100	50	90	14.1$\pm$0.1	13%
30	100	200	90	15.6$\pm$0.2	4%
30	100	500	90	15.6$\pm$0.4	4%
45	100	50	60	26.4$\pm$0.2	2%
45	100	200	60	25.4$\pm$0.3	2%
45	100	500	60	25.3$\pm$0.3	2%
45	100	1000	60	25.7$\pm$0.4	1%
22	34	102	22	15.8$\pm$0.2	1%

Supplementary Table 3 : Ray tracing results for $\Lambda_{\mathrm{B}} f$ of circular hole meshes.

Radius [nm]	Pitch p [nm]	Thickness t [nm]	$\Lambda_{\mathrm{B}} f$ [nm]	RNW error
425	1100	366	117$\pm$1.3	53%
5.5	34	22	22.9$\pm$0.1	35%

Supplementary Note 1: Porosity correction factor

Even in the absence of subcontinuum size effects, introducing porosity decreases thermal conductance *G*. To account for this bulk effect in interpreting measurements, several different conventions of a bulk porosity correction factor *f* have been taken in previous works when converting *G* to *k* . Yu *et al.*[4] assumed the effective cross-sectional area of the nanomesh to be the same as a nanowire array, resulting in $f=n/p_Y$. This method yields an effective upper bound on *k* due to the neglected area of the bridging necks. Tang *et al.*[5] used the experimental porosity ϕ to calculate a volumetric reduction $f= (1 - \phi)$, which yields a lower bound on *k*. El Kady *et al.*[3] and Jain et al.[6] used finite element method (FEM) simulations to obtain the porosity correction factor *f.* Among these conventions, using the FEM *f* factor is most meaningful because it allows direct comparison between diffusive theory predictions and experimental measurements of *k*.

To quantify the porosity correction factor *f*, we performed FEM simulations using COMSOL software for arrays of square holes and circular holes. The porosity correction factor can be readily obtained by the ratio of the thermal conductance *G* of mesh structures to *G* of completely filled structures, *i.e.* a homogenous plate with the same overall external dimensions but no holes. As shown in Supplementary Figure 1(a), the porosity correction factor for arrays of square holes and circle holes decreases as porosity increases and the porosity correction factor difference between arrays of square holes and circle holes is minimal (less than 3% for all porosities below 0.57). Next, we compared the FEM simulation results with the 2D Maxwell-Garnett effective medium model (EMM)[7] $f = (1 - \phi)/(1 + \phi)$ and find that results from FEM simulation and EMM are in excellent agreement (Supplementary Figure 1(a)).

We also computed the porosity correction factor *f* for rectangular holes with various aspect ratios using COMSOL, as shown in Supplementary Figure 1(b). It can be seen that *f* depends on aspect ratio and larger aspect ratios result in larger *f* for a given porosity. These FEM simulation results for rectangular holes agree well with a 2D EMM, suitably generalized for aligned elliptic cylinders[8], $f = (1 - \phi)/(1 + \phi * (p_y/p_x))$. In this work, we calculated *f* for our experiments using the measured porosity and 3rd order polynomial fitting between the FEM results for *f* as a function of porosity shown in Supplementary Figure 1(b).

Lastly, we illustrate the effect of different conventions for the porosity correction factor *f* on the reported conductivity *k* in Supplementary Figure 1(c). For the same experimentally measured quantities *G*, *L* and *A*, the choice of *f* can dramatically change the reported *k* value by a factor of 2 for typical nanomesh dimensions (here, the 100 nm by 100 nm mesh). Therefore, different *f* conventions must be taken into account when comparing data sets from multiple sources.

Supplementary Note 2: Details of BTE model

The inputs to the particle BTE model are the phonon dispersion and the scattering parameters. We chose an isotropic Born-van Karman (BvK) sine-type dispersion approximation $\omega = \omega_0 \sin\left(\frac{\pi}{2}\frac{\kappa}{\kappa_0}\right)$, where κ_0 is the cutoff wavevector set by the atomic number density and ω_0 is determined by the weighted speeds of sound such that $k(T)$ is exact in the low T boundary scattering limit[9]. Though silicon's dispersion is not isotropic, k of bulk and nanostructured silicon is often successfully modeled with an assumed isotropic dispersion[10], and the isotropic dispersion approximation is essential in interpreting the ray tracing results. The LA and two TA branches are lumped into one effective branch. Our previous studies showed that either an isotropic full dispersion model that accounts for six independent phonon branches[11] or the Born von Karman (BvK) model with lumped branches [9,10] adequately describes phonon transport in bulk and nanostructured silicon.

Different choices of dispersion can lead to different predictions of k reduction due to boundary scattering. For example, for the mesh with n=30 nm, t= 90 nm, and $p_x = p_y$ =100 nm, the BvK dispersion model for the pure silicon predicts around a 20% larger room temperature conductivity reduction than predicted by one first-principles approach[10]. The BvK model was selected because its predictions agree well with previous measurements of silicon nanostructures[10] from 20 to 300 K, and reduces to give the correct $k(T)$ in the boundary scattering regime by construction.

The Umklapp scattering was determined by a least-squares fit[10] of experimental $k(T)$. We use the Umklapp scattering rate $\tau_U^{-1} = P\omega^2 T \exp\left(-\frac{C_U}{T}\right)$ with $P = 1.53 * 10^{-19}$ s K^{-1} and $C_U = 144$ K. The impurity scattering rate for the literature silicon was $\tau_I^{-1} = C_I \omega^4$ with $C_I = 2.54 * 10^{-45}$ s^3. We fit the impurity scattering for our devices to the periodic and aperiodic data shown in Figure 2c and obtain $C_I = 1.96 * 10^{-44}$ s^3. This factor of 10 larger phonon impurity scattering rate appears reasonable given the literature silicon room temperature resistivity was 2000 Ω-cm (p-type)[12] and our boron-doped silicon resistivity is 14-22 Ω-cm, indicating larger dopant concentrations and scattering rates for our silicon. The impurity scattering modifies k by at most 15% at T=300 K, and for all T below 60 K the impurity correction to k is less than 5%.

We use Matthiessen's rule to combine ray tracing boundary scattering with frequency dependent intrinsic scattering when predicting $k(T)$. To test the validity of Matthiessen's rule for nanomesh structures, we also performed several ray tracing simulations that incorporate Umklapp scattering as well as boundary scattering. After a phonon is input to the domain or undergoes a scattering event, a free path is determined by drawing a random number ζ from a uniform distribution between 0 and 1. The phonon free path is then -$\Lambda_U \ln(\zeta)$, where Λ_U is the Umklapp scattering mean free path. If the free path is smaller than the distance to the next surface, an Umklapp scattering event occurs at the corresponding point within the domain, with the phonon's outgoing direction uniformly randomized among 4π sr. Supplementary Figure 2 compares the calculated Λ from the simulations explicitly including both Umklapp and boundary scattering with the predictions from Matthiessen's rule, for different input values of Λ_B/Λ_U. Both the AR=1 and AR=8 structures have $n = 30$ nm, t =90 nm, p=100 nm, and L =0.8 μm. The discrepancy between the points that include the coupled scattering and the dashed line showing

the Matthiessen's rule calculation is never larger than 7%, indicating that using Matthiessen's rule is not a major source of error in the high temperature analysis. Similarly good agreement between Matthiessen's rule and rigorously coupled scattering in nanostructures has also been observed in previous works[10,13].

Supplementary Note 3: Description of ray tracing computational implementation

We use a ray tracing method with Monte Carlo integration to find the average transmission coefficient $\langle\tau\rangle$ for the 3D nanomesh structures considering only boundary scattering. A phonon is launched at the device-lead contact with a randomly selected initial position. The azimuthal angle of injection is also uniformly random, while the polar angle is sampled from a $\cos\theta\sin\theta$ weighted distribution[14]. The phonon is propagated along a straight line to the nearest intersecting surface, and a uniform random number between 0 and 1 is drawn to determine the reflection angle. If the surface specularity parameter P is less than (greater than) the random number, then the particle is reflected diffusely (specularly). The ray tracing continues until the phonon exits the computational domain by reaching either thermalizing contact. For all results presented, at least 3*10^5 phonons were launched, and $\langle\tau\rangle$ is calculated as the number of transmitted phonons divided by number of total phonons injected.

As a measure of uncertainty, the standard deviations of $\langle\tau\rangle$ and thus k are estimated following a common subsampling method[15]. The phonons are uniformly distributed into J subsamples (here, J = 40). Each of these subsamples is now regarded as an independent measurement, and the difference between these subsamples provides an estimate of the variance of the total transmission coefficient $\langle\tau\rangle$. The mean transmission coefficients of the n subsamples $\langle\tau_n\rangle$ are calculated, and the variance σ^2 of $\langle\tau\rangle$ is found as

$$\sigma^2 = \frac{1}{J(J-1)}\sum_{n=1}^{J}[\langle\tau_n\rangle - \langle\tau\rangle]^2. \qquad \text{(Supplementary Equation 1)}$$

The computational domain is determined based on the symmetry of the structure. We take the global heat flow direction to be in-plane in $\hat{x}$. Since the experimental sample widths are much larger than the pitch, we consider only one unit cell in the in-plane direction $\hat{y}$ and apply fully specular boundary conditions on the periodic unit cell in $\hat{y}$ due to the mirror symmetry. The top and bottom surfaces of the film form two additional walls of the domain in the xy planes. The neck n between pores along $\hat{x}$ and $\hat{y}$ is always identical. When comparing the periodic and aperiodic mesh, we explicitly place distinct mesh pores along the length L of the device. For the aperiodic structure, n and p_y are held constant while p_x is varied randomly and uniformly by up to $\pm 20\%$ of the nominal spacing by drawing a different random number for each pore. We perform multiple trials with different aperiodic structures to achieve ensemble statistics.

For simulations where all structures of interest have a discrete translational symmetry along $\hat{x}$, we populate only one unit cell of surfaces to save computational time calculating the nearest collision. The yz mirror symmetry plane at the midpoint of the unit cell in $\hat{x}$ allows us to convert the periodic boundary condition to specular boundary conditions at $x = 0$ and $x = p_x$. We track the total distance the phonon has travelled along L and terminate the simulation when the phonon reaches either of the device-lead contact planes.

A characteristic behavior of diffusive transport is a mean free path Λ that saturates for long L. The mean free path Λ calculated from the transmission coefficient $\langle\tau\rangle$ describes the combined effects of diffusive nanomesh boundary scattering and a length-independent ballistic resistance (which can be represented through a transmission coefficient τ_{ball}) that depends on the configuration of the device-contact connection. To find the intrinsic Λ_{B}, defined as $\lim_{L\to\infty}(\Lambda)$, from finite-$L$ simulations, we follow the technique of previous works[16] and sum the ballistic and diffusive scattering in parallel in a Matthiessen's rule-type approximation, resulting in

$$\frac{L}{\Lambda}=\frac{1}{\tau_{\mathrm{ball}}}+\frac{L}{\Lambda_{\mathrm{B}}}. \qquad \text{(Supplementary Equation 2)}$$

We calculate Λ as a function of L and use a weighted least-squares fitting to Supplementary Equation 2 to determine Λ_{B}. (If ballistic behavior is important even for large L, as is the case for transport in a fully specular nanowire or a diffuse thin film in the in-plane direction in the absence of Umklapp scattering[17], then Λ does not converge with L ; this is not the case here.) Supplementary Figure 3 illustrates this procedure for the P=0 scenario from Figure 2 in the main text.

Supplementary Note 4: Ray Tracing Validation for Nanomesh Thermal Conductivity

As an additional validation of our modeling technique, we compare the ray tracing simulation predictions against published computational results for a silicon nanomesh[1]. The literature results were obtained using a variance-reduced Monte Carlo (VRMC) algorithm to solve the BTE while including both boundary and intrinsic scattering in the 3D diffuse nanomesh. We performed ray tracing simulations using the same dimensions and specularity to obtain Λ_{B} for a mesh with square holes ($p=34$ nm, $n=23$ nm, $t=22$ nm) and a mesh with circular holes ($p=34$ nm, diameter d =11 nm, $t=22$ nm). For the purpose of this comparison only, we use the same Si [100] dispersion and intrinsic scattering times as in the original work[1] to make a fair comparison between numerical predictions. We note that the plotted quantity for the mesh in the reference[1] is $k_{\mathrm{eff}}=kf=GL/A$, and we use the EMM result that matched well with our FEM analysis, $f=(1-\phi)/(1+\phi)$, for both circular and square holes. As shown in Supplementary Figure 4, the ray tracing and the VRMC predictions for $k_{\mathrm{eff}}(T)$ show excellent agreement within 3% for all temperatures for the circular and square holes, validating our ray tracing code. The small remaining discrepancy between techniques may be due to our use of Matthiessen's rule or slight differences in the dispersion fits. A benefit of using a ray tracing approach as compared to solving the BTE with a known mean free path spectrum as an input is that in the ray tracing, the material-independent Λ_{B} is determined once and for all for a given geometry and surface specularity. Once Λ_{B} is found from the ray tracing, different isotropic dispersions, intrinsic scattering rates, or temperatures are then easily incorporated using Matthiessen's rule without requiring the additional computationally expensive simulations of a BTE solver. We note that Λ_{B} could also be found by solving the BTE assuming that all phonons have the same mean free path. In addition, detailed BTE solvers can also obtain the full temperature profile in addition to the heat flux.

Supplementary Note 5: Porosity Calculation

The hole shapes are neither perfect squares nor perfect circles due to the complexity of microfabrication processes. As a result, quantification of porosity is crucial for obtaining the porosity correction factor, which is required to finally convert experimentally measured thermal conductance to thermal conductivity. Thus, we performed image processing on SEM images of Si nanomesh structures using MATLAB to obtain the porosity. First, we convert SEM images to binary images using a grayscale intensity threshold. Then, the pixels corresponding to the empty and the filled state are each counted on the binary images. The porosity ϕ can be readily obtained from ϕ = (number of pixels of empty state)/(total number of pixels). The obtained porosity is used to calculate the porosity correction factor, as described in Supplementary Note 1.

Supplementary Note 6: Experimental Uncertainty

We start with the thermal conductivity expression $k = \frac{GL}{fwt}$ (Eq. 1) to estimate the uncertainty of the experimental data using propagation of error. The relative uncertainty of *k* can be written as

$$\frac{\delta k}{k} = \sqrt{\left(\frac{\delta G}{G}\right)^2 + \left(\frac{\delta L}{L}\right)^2 + \left(\frac{\delta f}{f}\right)^2 + \left(\frac{\delta w}{w}\right)^2 + \left(\frac{\delta t}{t}\right)^2}. \quad \text{(Supplementary Equation 3)}$$

The nanomesh conductance *G* is determined[18] by measuring the temperature rise of the heating membrane ΔT_h and the temperature rise of the sensing membrane ΔT_s to an input heating power *Q*. Since $\Delta T_h >> \Delta T_s$ in our experiments, the relative uncertainty of *G* is

$$\frac{\delta G}{G} = \sqrt{\left(\frac{\delta Q}{Q}\right)^2 + \left(\frac{\delta(\Delta T_\mathrm{s})}{\Delta T_\mathrm{s}}\right)^2 + 2\left(\frac{\delta(\Delta T_\mathrm{h})}{\Delta T_\mathrm{h}}\right)^2}. \quad \text{(Supplementary Equation 4)}$$

The dominant term of $\delta G/G$ is $\frac{\delta(\Delta T_\mathrm{s})}{\Delta T_\mathrm{s}}$, which is estimated to be 1~3 %. The length *L*, width *w*, and thickness *t* are measured by SEM. While $\delta L/L$ and $\delta w/w$ are negligible, $\delta t/t$ is ~4 % considering the uncertainty (~3 nm) of the SEM measurement. *f* is estimated as described previously in Supplementary Notes 1 and 5, and due to the uncertainty in the SEM measurement, $\delta f/f$ is ~4 %. Consequently, $\delta k/k$ is estimated to be ~6 % by error propagation.

Supplementary Note 7: Periodic and Aperiodic Thermal Conductivity

A main conclusion of this work is that the particle BTE model predicts very similar *k* for the periodic and aperiodic nanomeshes that are experimentally measured. We presented ray tracing results in the main text (Fig. 2d) showing the periodic and aperiodic Λ as a function of *L* for two surface specularities, *P*=0 and *P*=0.8. Supplementary Figure 5 shows the periodic/aperiodic thermal conductivity ratio $k_\mathrm{p}/k_\mathrm{ap}$ in the boundary scattering regime as a

function of specularity P for nanomeshes with the same dimensions in Figure 2 and fixed L=1.6 µm. For very large specularities (P>0.9), the periodic conductivity is significantly larger than the aperiodic conductivity. We attribute this result to a "critical angle" effect: for the periodic structures, phonons incident at critical angles can be specularly reflected forward through the nanomesh for many periods before being backscattered, as illustrated in the specular cartoon in Supplementary Figure 5. Introducing aperiodicity can backscatter these critical angle phonons due to the shifted positions of the holes. Of course, adding the aperiodicity can also cause some phonons who were previously not transmitted in the periodic structure to transmit; however, the increased Λ for periodic structures at P=1 indicates that the net effect of the aperiodicity is to increase backscattering in this scenario.

These ray tracing results reveal that at very low temperature (<5 K) where the dominant phonon wavelengths are very large compared to surface roughness so that surfaces are well approximated as fully specular, the particle model also predicts that k_{p} and k_{ap} can differ. But this clearly has nothing to do with coherence effects on the phonon dispersion relation. Thus, in this regime great care must be taken to properly identify the coherent contributions to thermal transport, since differences cannot be simply attributed to wave effects. Lastly, we note that our experimental trends and comparisons thoughout the main text are well described by the P=0 ray tracing predictions. Such fully diffuse behavior in our temperature range is expected from specularity model predictions such as Ziman's commonly used expression[19] assuming surface roughness >1 nm, as well as previous measurements of silicon nanostructures[20,21].

Supplementary Note 8: Backscattering definition

Our definition of the backscattering fraction as the fraction of the total boundary scattering events that change the $\hat{x}$ component of the phonon velocity from positive to negative for the phonons emitted from the hot reservoir differs slightly from previous discussions of backscattering in the literature. In the previous work by Ravichandran and Minnich[1], backscattering is determined by whether an energy packet, which is emitted at the entrance of the repeated unit cell and which has one or more boundary scattering events within the cell, exits the repeated unit cell at the far end or returns to the entrance. Moore *et al*[22] use the term backscattering to describe the heat flux diffusely reflected from a single sawtooth on a nanowire surface. Though our definition is similar to these two scenarios, our method counts each boundary scattering event individually rather than summarizing the effect of one periodic unit cell or one sawtooth on the flux. In our definition, the choice of periodic unit cell has no influence on the backscattering fraction calculation, which is desirable because there are an infinite number of valid choices of primitive unit cell.

An alternate "both directions" definition of backscattering would consider both $+\hat{x}$ to $-\hat{x}$ and $-\hat{x}$ to $+\hat{x}$ boundary scattering events for phonons emitted from the hot reservoir as backscattering, since the phonon leaves the boundary in a different direction than it entered. The backscattering fraction would then be defined as the number of backscattering events divided by the number of total boundary scattering events for phonons emitted from the hot

reservoir. Due to the high symmetry of the nanomesh, the fraction of boundary scattering events in which phonons heading in $+\hat{x}$ are scattered into the $-\hat{x}$ direction is identical to the fraction of boundary scattering events in which phonons heading in $-\hat{x}$ are scattered into the $+\hat{x}$ direction for long nanomeshes. For short nanomeshes, edge effects can result in backscattering fraction asymmetries; for all results shown here, we have verified that the backscattering fraction changes by less than 5% (*e.g.* from 0.5 to 0.5025) with an increase of a factor of 2 in length. Therefore all numerical results in the main text are unchanged whether the backscattering fraction definition of the main text is used or the "both directions" definition discussed here is used.

Lastly, we note that the transmittance $\langle\tau\rangle$ calculated in the ray tracing fundamentally quantifies the total backscattering of the device including multiple backscattering effects over the entire length, since 1-$\langle\tau\rangle$ is the probability that a phonon injected at the entrance returns to the entrance due to any combination of backscattering events. Therefore conductance *G* in the boundary scattering regime is itself a direct measure of the total device backscattering, and the backscattering capability of different devices can be compared using the product *GL/A.*

Supplementary Note 9: Rectangular Nanowire Equivalent Conductance

Figure 4 in the main text showed that the ray tracing for diffuse surfaces predicts that conductance *G* is independent of pitch aspect ratio. Since *G* is proportional to $\Lambda_{\mathrm{B}}f$ in the boundary scattering regime, $\Lambda_{\mathrm{B}}f$ of a mesh with square or rectangular holes and $\Lambda_{\mathrm{B}}f$ of a nanowire array (large aspect ratio structure) with the same n and t are similar for the nanomesh dimensions considered in this work. This indicates that the boundary scattering mean free path of a rectangular nanowire $\Lambda_{\mathrm{B,RNW}}$ can be used to estimate the mean free path of the nanomesh $\Lambda_{\mathrm{B,mesh}}$ using the relation $\Lambda_{\mathrm{B,mesh}}f = \Lambda_{\mathrm{B,RNW}}n/p_y$. McCurdy, Maris, and Elbaum[2] derived the analytical solution for $\Lambda_{\mathrm{B,RNW}}$ of a long diffuse rectangular bar with height h, width w and aspect ratio $r = h/w$ as

$$\Lambda_{\mathrm{B,RNW}} = \frac{\sqrt{hw}}{4}\Big[3r^{-\frac{1}{2}}\ln\left(r^{-1}+\sqrt{1+r^{-2}}\right) + 3r^{\frac{1}{2}}\ln\left(r+\sqrt{1+r^{2}}\right) - r^{\frac{1}{2}}\sqrt{1+r^{2}} - r^{-\frac{1}{2}}\sqrt{1+r^{-2}} + r^{\frac{3}{2}} + r^{-\frac{3}{2}}\Big]\,. \qquad \text{(Supplementary Equation 5)}$$

We also verified Supplementary Equation 5 by numerically integrating the arbitrary nanowire cross-section equation for Λ_{B} presented in Ziman[19] for different aspect ratios. Supplementary Figure 6 plots Supplementary Equation 5 for $\Lambda_{\mathrm{B,RNW}}/w$ as a function of r. Supplementary Equation 5 is invariant upon interchange of the labels h and w, so we only need consider $r \geq 1$. For r near one, a common approximation[23–25] is to take $\Lambda_{\mathrm{B,RNW}} \approx 1.12\sqrt{hw}$ in analogy with the square nanowire of diameter D result $\Lambda_{\mathrm{B,SNW}} = 1.12D$. This error in the $1.12\sqrt{hw}$ approximation is less than 5% for $r < 2.5$.

Surprisingly, we see that the large r limit,

$$\Lambda_{\mathrm{B,RNW,LR}} = \frac{3w}{4}\left[\ln 2r + \frac{1}{3r} + \frac{1}{2}\right] \quad \text{(Supplementary Equation 6)}$$

is within 3% of the exact result for all $r > 1$ and more accurate than the small r limit for $r > 1.5$. This large r limit is reminiscent of the logarithmic scaling[17] for in-plane conductivity of a thin film with thickness t and Umklapp scattering Λ_{U}, for which $\Lambda_{\mathrm{in-plane}} = \frac{3t}{4}\ln\frac{\Lambda_{\mathrm{U}}}{t}$ when $\Lambda_{\mathrm{U}} \gg t$. Therefore, when modeling boundary scattering in rectangular nanowires, Supplementary Equation 6 may be used as a convenient and accurate approximation to Supplementary Equation 5 over all r.

Supplementary Note 10: Tabulated ray tracing results for diffuse surfaces

For completeness, we present numerical ray tracing results for boundary scattering in periodic structures with all diffuse surfaces in the long-length limit. For example, the value of $\Lambda_{\mathrm{B}} = 44.2$ nm found from the diffuse surface simulations in Fig. 2d corresponds to first row's value of $\Lambda_{\mathrm{B}} f = 15.2$ nm in Supplementary Table 1. For all results shown here, the neck *n* between holes is the same in both in-plane directions $\hat{x}$ and $\hat{y}$. We also calculate the percentage error in the equivalent nanowire approximation (discussed in Supplementary Note 9) $\Lambda_{\mathrm{B}} f = \Lambda_{\mathrm{B,RNW}}(n/p_y)$, where $\Lambda_{\mathrm{B,RNW}}$ is calculated from the known analytical solution[2] for a rectangular nanowire with the same *n* and *t* as the mesh. Supplementary Table 1 shows results for a mesh with square holes arranged in a square array, Supplementary Table 2 shows results for a mesh with rectangular holes in a rectangular array, and Supplementary Table 3 shows results from a square array of circular holes. We note that the rectangular nanowire assumption is generally accurate at the 10% level for square and rectangular holes, but that using the smallest dimension between circular pores as an effective nanowire n is a poor predictor of the circular mesh conductance, as shown in Supplementary Table 3.

Supplementary Note 11: Phonon backscattering

Figure 4(d) in the main text showed that the increase in backscattering fraction for the nanomesh did not affect the conductance *G*, which is directly proportional to $\Lambda_{\mathrm{B}} f$ in the boundary scattering regime. There are several possible hypotheses we considered to describe the apparent lack of correlation between backscattering fraction and conductance for diffuse surfaces. For example, in small *AR* meshes, the lateral necks (*n*) between pores in $\hat{x}$ are relatively large compared to the hole length (p_x-n) along $\hat{x}$, which could feasibly provide additional channels for phonons to transmit forward instead of hitting a hole in a large *AR* nanomesh. This argument would predict that transmitted phonons hit fewer surfaces in small *AR* meshes: even though more of the boundary scattering events result in backscattering and are efficient at reducing heat transfer, there may be fewer scattering events overall. Another hypothesis is that multiple backscattering effects dominate. Phonons which backscatter once inside the mesh necks can scatter again off of the opposite wall before exiting the mesh in the

original direction, or indeed may backscatter many times within the necks before reemerging. The multiple backscattering argument predicts that phonons transmitted through short *AR* meshes will hit more surfaces on average than phonons transmitted through the long *AR* meshes.

Supplementary Figure 7 shows the average number of boundary scattering collisions per transmitted phonon $\langle N_{\mathrm{c}} \rangle$, normalized by the *AR*=10 value $\langle N_{\mathrm{c},AR=10} \rangle$. The inset shows $\langle N_{\mathrm{c}} \rangle$ as a function of length L for different aspect ratios, indicating that the ratio $\langle N_{\mathrm{c}} \rangle / \langle N_{\mathrm{c},AR=10} \rangle$ is independent of L. $\langle N_{\mathrm{c}} \rangle$ is larger for smaller aspect ratios, meaning that phonons transmitted through small *AR* meshes on average hit more surfaces than phonons transmitted through large *AR* meshes. This result supports the multiple backscattering interpretation.

Supplementary Note 12: Particle model explanation for previous experimental result using ray tracing

A recent experimental work[3] measured k of silicon nanomeshes with circular holes (p=1100 nm, t=366 nm, n=250 nm, and various diameters) and suggested that coherence effects arising from the mesh periodicity are important for heat transfer at 300 K. Here, we use ray tracing simulations to compute rigorous incoherent model predictions for these geometries assuming diffuse scattering, and find that the particle model also predicts the experimental trend previously attributed to phonon wave effects.

The experiment[3] was designed to manipulate the phononic band structure by interpenetrating an additional periodic array of small circular holes (radius r_{s}=102.5 nm) within the larger periodic mesh. The work calculates a modified dispersion relation for sub-10 GHz phonons due to changes in this secondary periodicity for five meshes with different interpenetrated arrays. We note that 10 GHz is relatively low energy compared to the characteristic thermal frequency at 300 K, estimated as $f_{\mathrm{th}} = \frac{k_{\mathrm{b}} T}{h} \approx 6{,}250$ GHz, where h is Planck's constant and k_{b} is Boltzmann's constant. Two trends in the experimental results were interpreted as suggestive of room temperature coherence effects. First, the measured k_{mesh} normalized by a measured k_{tf} of a film of the same thickness was smaller than a "lower-bound" prediction of a BTE model using an assumed $\Lambda_{\mathrm{B}} = n$. Second, the experimental k_{mesh} trend showed a stronger decrease with increasing porosity than predicted by a BTE model with constant Λ_{B} between structures. A crucial approximation in the reported BTE analysis is that Λ_{B} was essentially unchanged between the different meshes even though additional holes have been introduced.

We performed ray tracing simulations using the geometries and dimensions of the five reported mesh structures. Contrary to the previous assumption[3], we find that Λ_{B} decreases monotonically with the number of additional small holes, and that the decrease in Λ_{B} from the original supercell mesh and the "1x1" mesh with the highest density of small holes is around 20% (from $\Lambda_{\mathrm{B}} = 320$ nm to $\Lambda_{\mathrm{B}} = 256$ nm). We use these Λ_{B} values as inputs to the BvK model using Matthiessen's rule with the same silicon scattering parameters used in the main text here, to find k_{mesh} at 300 K using our definition of k from Eq. 1 in the main text. All surfaces are taken fully diffuse. To focus on the BTE model predictions for the change in k_{mesh} with additional

holes, we normalize the simulation and experimental values by their respective supercell conductivity k_{SC} (see leftmost schematic of Supplementary Figure 8). We present results using the BvK model for clarity since the six different models analyzed in Yang and Dames[10] yield $k_{\mathrm{mesh}}/k_{\mathrm{SC}}$ that differ by no more than 4%.

Supplementary Figure 8 shows $k_{\mathrm{mesh}}/k_{\mathrm{SC}}$ as measured in the experiment[3] and as predicted here from the ray tracing and BTE model described in the preceding paragraph. The labeling notation follows the original work, and the experimental fractional percentage uncertainty in $k_{\mathrm{mesh}}/k_{\mathrm{SC}}$ is taken to be the same as the reported percentage uncertainty[3] in $k_{\mathrm{mesh}}/k_{\mathrm{tf}}$. In the BTE model, the reduced k_{mesh} with increasing small hole density is due exclusively to increased boundary scattering because the BTE model uses bulk dispersion and bulk intrinsic scattering. This result shows that the BTE model predicts the observed experimental trend in $k_{\mathrm{mesh}}/k_{\mathrm{SC}}$ once boundary scattering is rigorously considered, without any appeal to coherent effects.

Supplementary References:

1. Ravichandran, N. K. & Minnich, A. J. Coherent and incoherent thermal transport in nanomeshes. *Phys. Rev. B* **89,** 205432 (2014).

2. McCurdy, a., Maris, H. & Elbaum, C. Anisotropic Heat Conduction in Cubic Crystals in the Boundary Scattering Regime. *Phys. Rev. B* **2,** 4077–4083 (1970).

3. Alaie, S. *et al.* Thermal transport in phononic crystals and the observation of coherent phonon scattering at room temperature. *Nat. Commun.* **6,** 7228 (2015).

4. Yu, J.-K., Mitrovic, S., Tham, D., Varghese, J. & Heath, J. R. Reduction of thermal conductivity in phononic nanomesh structures. *Nat. Nanotechnol.* **5,** 718–721 (2010).

5. Tang, J. *et al.* Holey silicon as an efficient thermoelectric material. *Nano Lett.* **10,** 4279–4283 (2010).

6. Jain, A., Yu, Y.-J. & McGaughey, A. J. H. Phonon transport in periodic silicon nanoporous films with feature sizes greater than 100 nm. *Phys. Rev. B* **87,** 195301 (2013).

7. Hashin, Z. & Shtrikman, S. A Variational Approach to the Theory of the Effective Magnetic Permeability of Multiphase Materials. *J. Appl. Phys.* **33,** 3125 (1962).

8. Mityushev, V. Conductivity of a two-dimensional composite containing elliptical inclusions. *Proc. R. Soc. A Math. Phys. Eng. Sci.* **465,** 2991–3010 (2009).

9. Dames, C. & Chen, G. Theoretical phonon thermal conductivity of Si/Ge superlattice nanowires. *J. Appl. Phys.* **95,** 682 (2004).

10. Yang, F. & Dames, C. Mean free path spectra as a tool to understand thermal conductivity in bulk and nanostructures. *Phys. Rev. B* **87,** 035437 (2013).

11. Pop, E., Dutton, R. W. & Goodson, K. E. Analytic band Monte Carlo model for electron transport in Si including acoustic and optical phonon dispersion. *J. Appl. Phys.* **96,** 4998–5005 (2004).

12. Glassbrenner, C. J. & Slack, G. a. Thermal conductivity of silicon and germanium from 3K to the melting point. *Phys. Rev.* **134,** 1058 (1964).

13. Hori, T., Shiomi, J. & Dames, C. Effective phonon mean free path in polycrystalline nanostructures. *Appl. Phys. Lett.* **106,** 171901 (2015).

14. Press, W. H., Teukolsky, S. A., Vettering, W. T. & Flannery, B. P. *Numerical Recipes in C: The Art of Scientific Computing, Second Edition*. (1992).

15. Modest, M. *Radiative Heat Transfer*. (Academic Press, 2003).

16. Heron, J.-S., Bera, C., Fournier, T., Mingo, N. & Bourgeois, O. Blocking phonons via nanoscale geometrical design. *Phys. Rev. B* **82,** 155458 (2010).

17. Fuchs, K. & Mott, N. F. The conductivity of thin metallic films according to the electron theory of metals. *Math. Proc. Cambridge Philos. Soc.* **34,** 100 (1938).

18. Shi, L. *et al.* Measuring Thermal and Thermoelectric Properties of One-Dimensional Nanostructures Using a Microfabricated Device. *J. Heat Transfer* **125,** 881 (2003).

19. Ziman, J. M. *Electrons and Phonons*. (1960).

20. McConnell, A. D. & Goodson, K. E. in *Annual review of heat transfer* 129–168 (2005).

21. Marconnet, A. M., Asheghi, M. & Goodson, K. E. From the Casimir Limit to Phononic Crystals: 20 Years of Phonon Transport Studies Using Silicon-on-Insulator Technology. *J. Heat Transfer* **135,** 061601 (2013).

22. Moore, A. L., Saha, S. K., Prasher, R. S. & Shi, L. Phonon backscattering and thermal conductivity suppression in sawtooth nanowires. *Appl. Phys. Lett.* **93,** 083112 (2008).

23. Heron, J. S., Fournier, T., Mingo, N. & Bourgeois, O. Mesoscopic size effects on the thermal conductance of silicon nanowire. *Nano Lett.* **9,** 1861–1865 (2009).

24. Lü, X., Chu, J. H. & Shen, W. Z. Modification of the lattice thermal conductivity in semiconductor rectangular nanowires. *J. Appl. Phys.* **93,** 1219 (2003).

25. Hippalgaonkar, K. *et al.* Fabrication of microdevices with integrated nanowires for investigating low-dimensional phonon transport. *Nano Lett.* **10,** 4341–8 (2010).
